# A Study of the Efficacy and Safety of Aerobic Exercise Training in Pulmonary Arterial Hypertension (the Saturday Study): Protocol for a Prospective, Randomized, and Controlled Trial

**DOI:** 10.3389/fmed.2022.835272

**Published:** 2022-04-05

**Authors:** Rong Jiang, Lan Wang, Ping Yuan, Qin-Hua Zhao, Su-Gang Gong, Jing He, Hong-Ling Qiu, Ci-Jun Luo, Rui Zhang, Ting Shen, Meng-Yi Zhan, Yu-Mei Jiang, Fa-Dong Chen, Jin-Ming Liu, Yu-Qin Shen

**Affiliations:** ^1^Department of Cardio-Pulmonary Circulation, Shanghai Pulmonary Hospital, Tongji University School of Medicine, Shanghai, China; ^2^Department of Cardiac Rehabilitation, Tongji Hospital, Tongji University School of Medicine, Shanghai, China; ^3^Department of Cardiology, Tongji Hospital, Tongji University School of Medicine, Shanghai, China

**Keywords:** pulmonary arterial hypertension, exercise training, rehabilitation, exercise intolerance, cardiac magnetic resonance imaging

## Abstract

**Background:**

Patients with pulmonary arterial hypertension (PAH) have reduced exercise capacity and poor quality of life. Exercise-based rehabilitation in PAH results in clinically relevant improvements in exercise capacity and hemodynamics. To clarify the mechanism, we will evaluate the effect of aerobic exercise training rehabilitation on right ventricular (RV) remodeling and function as determined measured by cardiac magnetic resonance imaging (CMR).

**Methods:**

We will conduct a 26-week multicenter randomized controlled trial. Patients on stable and unchanged PAH-targeted medication are randomly assigned (1:1) to the control and training groups. The primary endpoint is the RV stroke volume (RVSV) change from baseline to Week 26, determined by CMR. Comprehensive RV function is also performed using CMR. Other characteristics of the RV and left ventricle, World Health Organization functional class, 6-min walk distance, and N-terminal pro-B-type natriuretic peptide are included in secondary endpoints. We also investigate the proteomic, metabolomic, and transcriptomic changes after exercise training as exploratory endpoints.

**Ethics and Dissemination:**

The study and protocol were approved by the Ethics Committee of Shanghai Pulmonary Hospital (Approved No. of ethics committee: L20-17). The results will be disseminated at medical conferences and in journal publications. All participants will sign written informed consent.

**Trial Registration Number:**

ChiCTR2000031650.

## Introduction

Pulmonary arterial hypertension (PAH) is characterized by progressive remodeling of the pulmonary vasculature, which results in limited exercise capacity, progressive increase in breathlessness and right ventricular remodeling, and eventual death (1). Advances in PAH-targeted medications have significantly improved prognosis, and other therapies need to be further explored in order to improve exercise capacity and quality of life (QoL) (2). There is strong evidence that exercise training improves cardiorespiratory fitness, functional status, and clinical outcomes of patients with other heart or lung diseases (3, 4). There was a time when PAH patients were actively discouraged from exercise rehabilitation because of fear of exercise risk. However, exercise training has recently shown beneficial effects as add-on to PAH-targeted treatment in different etiologies of pulmonary hypertension (PH), such as IPAH (3, 5–8), inoperable chronic thromboembolic PH (CTEPH) (4), and associated PAH in connective tissue diseases (9).

Supervised rehabilitation, including exercise training have been proved to improve pulmonary hemodynamics, exercise capacity, QoL, and possibly RV function in patients with PH (Class I, A) (5–15). Currently, most exercise training trials published have focused on exercise capacity of patients with PH. The European Respiratory Society statement on exercise training and rehabilitation acknowledges that gaps in knowledge exist (15). Among them, the mechanisms of improved exercise capacity and hemodynamics by exercise training in PAH remain incompletely understood.

Increased cardiac output may be explained by a decreased RV afterload and direct training effect on heart. Therefore, further research on the effects of exercise training in PAH should consider the endpoints of RV properties.

Cardiac magnetic resonance imaging (CMR) is the gold standard for non-invasive assessment of RV function. Images of slices in any plane can be obtained with a very good spatial and temporal resolution. This translates into a low variability of inter-study and inter-rater CMR assessments which allows a reduced sample size for clinical trials compared to other imaging tools (16).

Therefore, a randomized controlled trial (RCT) comparing aerobic exercise training as an adjunctive therapeutic strategy is being conducted in China. This study aims to evaluate the effect of aerobic exercise training on right ventricular properties assessed by CMR in patients with PAH.

## Methods And Analysis

### Study Design and Setting

**Figure 1:**
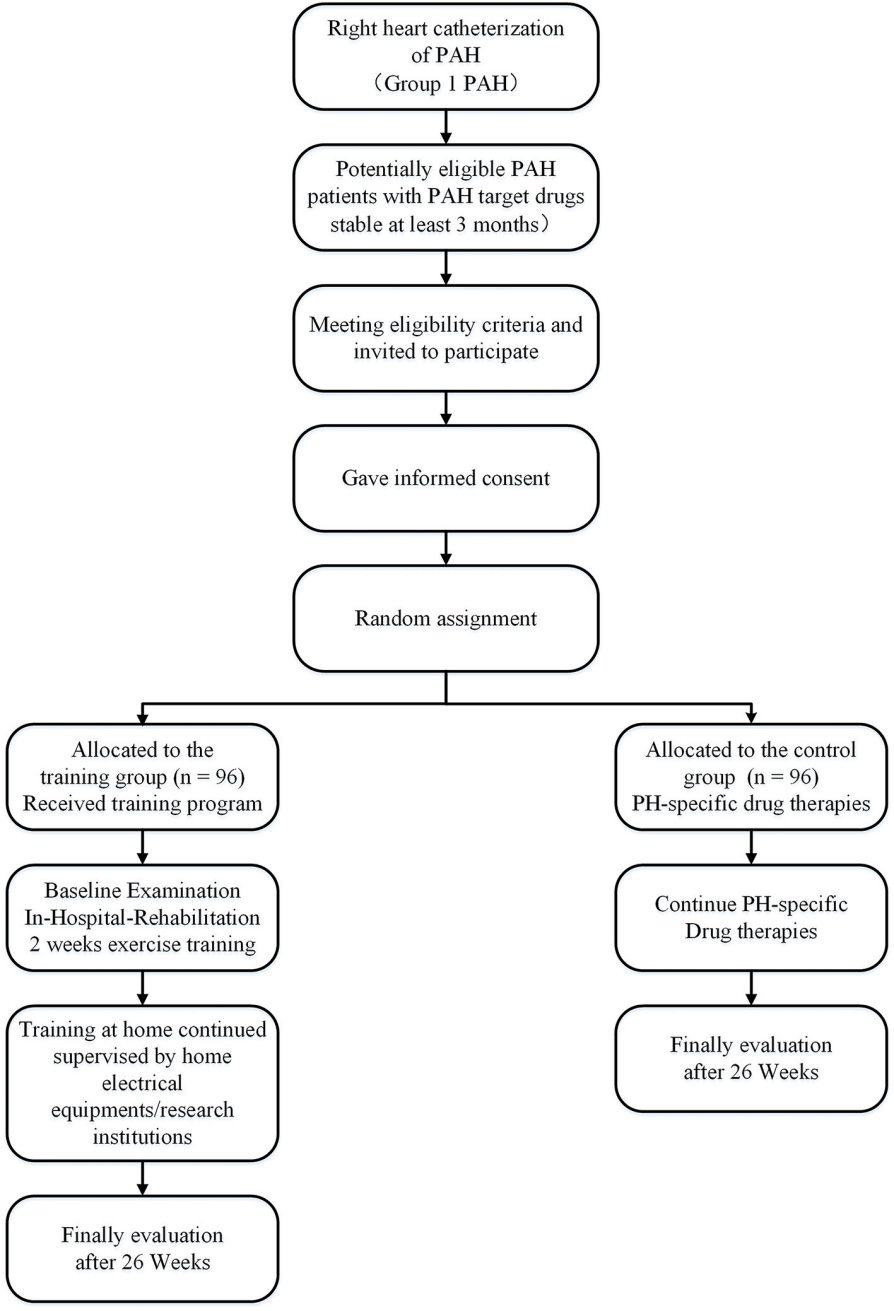
Flow diagram of study recruitment and randomization.

The study of the efficacy and safety of aerobic exercise training in PAH (the Saturday study) will recruit subjects from 1 June 2020 to 1 June 2023. The study is a 26-week multicenter, prospective, and RCT. Subjects willing to consider enrollment will be consented. Patients with PAH stable on PAH-targeted drugs for at least 3 months prior to inclusion are randomly assigned to the control and training groups. Medication remains unchanged during the study period (Figure 1). We will use the SPIRIT checklist and TIDiER checklist when writing our report (Supplementary Materials 1, 2) (17, 18). All centers will follow a standardized protocol, including rehabilitation, diagnosis, and evaluation of PAH, as shown in Table 1.

**Table 1:** Frequency, intensity, time, and type of exercise training.

	**In-hospital**	**Home**
Frequency	3 days per week for 2 weeks	3 days per week for 10 weeks
Intensity	in AT heart rate	in AT heart rate
Time	30 min/section	30 min/section
Type	Walking, cycling	walking, cycling

*AT, anaerobic threshold*.

### Eligibility Criteria

#### Inclusion Criteria

(a) Group 1 PAH (idiopathic, heritable, drugs, connective tissue diseases, or all kinds of congenital heart diseases) based on the diagnostic criteria of the 6th World Symposium on Pulmonary Hypertension (19); (b) age ≥ 18 or <60 years, (c) World Health Organization functional class (WHO FC) II or III, (d) stable and compensated under PAH-targeted medications, diuretics, and supplemental oxygen for at least 3 months; (e) willing to sign written informed after full explanation of this study; (f) 6-min walking distance (6 MWD) ≥ 200 m.

#### Exclusion Criteria

(a) Group 2–5 PH groups in accordance with diagnostic criteria (19).

(b) Uncontrolled hypertension (systolic blood pressure > 180 mm Hg or diastolic blood pressure > 110 mm Hg), unstable arrhythmias, angina or myocardial infarction, stroke or transient ischemic attach (within 3 months) (20).

(c) Inability to mobilize independently.

(d) Pulmonary embolism or deep vein thrombosis in the last 6 months prior to screening or without regular or standard anticoagulation.

(e) Neuromuscular disorders limiting rehabilitation.

(f) Unsuitable for participation by researchers, such as unsafe in exercise training or poor compliance.

### Recruitment and Consent

The potentially eligible subjects were provided informed consent documents for a comprehensive explanation of the trial. Participants will be identified by the clinical or rehabilitation physician, *via* screening strategies from the databases of our own PAH patients. As a general rule, patients with definitive diagnosis of Group 1 PAH who are willing to consider enrolment will be asked to provide consent.

### Rehabilitation Program With Aerobic Exercise Training

**Figure 2:**
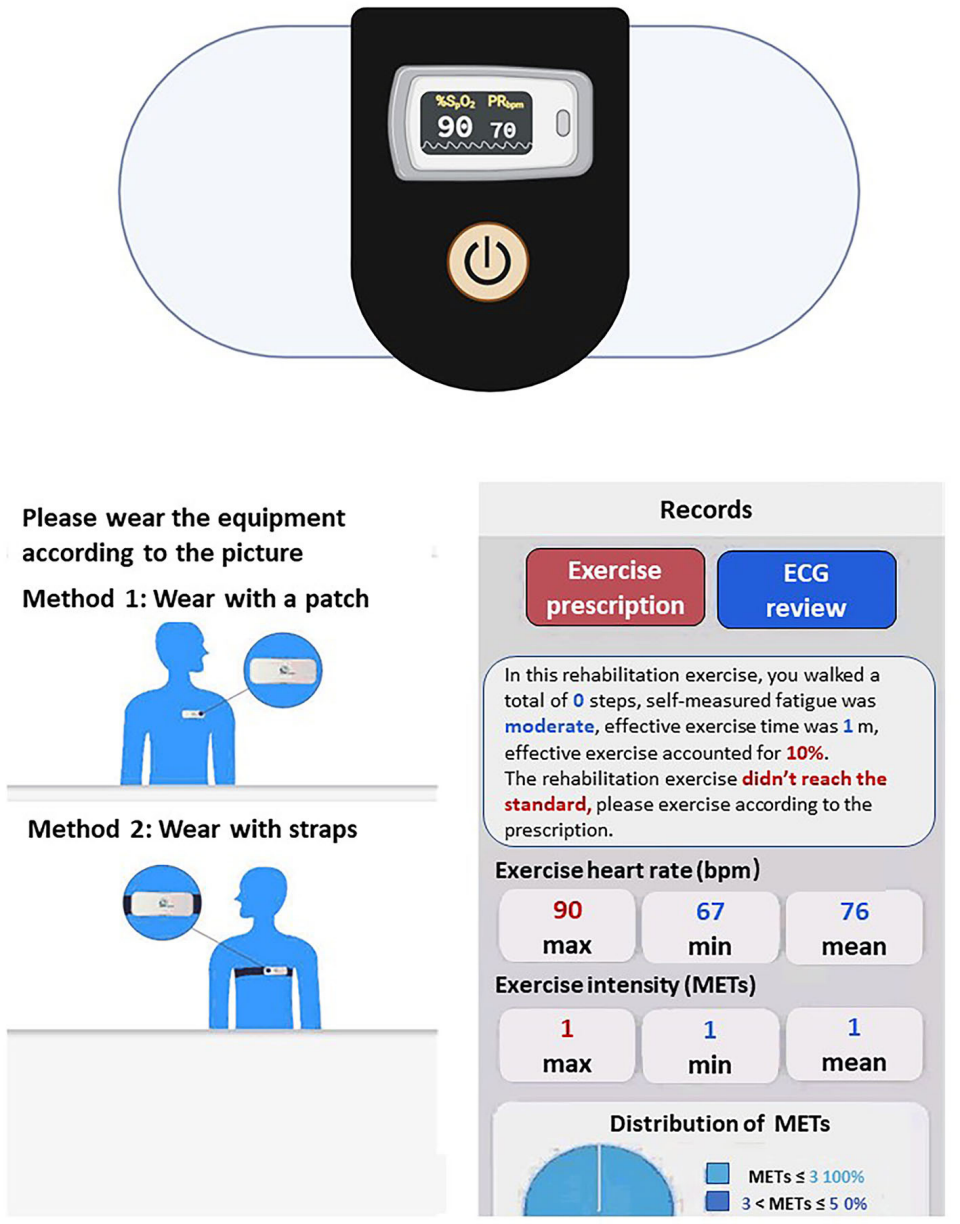
The screenshot for the portable electrical equipment and the *Xinankang* app for patients (English version). Instructions on how to wear a close-fitting the portable electrical equipment and rehabilitation record details including metabolic equivalent, heart rate and exercise prescription, etc.

Exercise training is started in-the hospital for 2 weeks. Patients spend 30 min per session 3 days/week. A cardiopulmonary exercise test prior to enrolment was performed to determine the anaerobic threshold (AT). We perform interval cycle ergometer training at workloads of 1 min before the AT. The training intensity was adjusted by oxygen saturation (SaO_2_), heart rate (HR), and blood pressure monitored throughout the training. Patient receive oxygen supply throughout the training when SaO_2_ falls below 90% during exercise. Patients who were on daily oxygen before enrollment remain on oxygen. The exercise training includes two aerobic exercise training sessions, namely, 2-week supervised exercise training in the hospital and 10-week training at home. The home-based program is walking with exercise intensity at an AT of 30 min/day for 3 days a week for 10 weeks. Detailed FITT principle is shown in Table 1. In our study protocol, we choose AT intensity because AT intensity stands for aerobic exercise, which can avoid the accumulation of lactic acid in the body, avoid excessive activation of sympathetic nerve, excessive myocardial oxygen consumption, or arrhythmia. The AT intensity is expressed by the HR or the number of steps (the speed of the AT divided by the step distance). Patients in this group at home are managed by the *Xinankang* home electrical equipment system together with PAH experts, physiatrists, and physiotherapists. The home system consists of the portable electrical equipment and *the Xinankang* app for patients to upload their health data, and communicate with the *Xinankang* app uploaded by physiatrists and physiotherapists. This app also reminds participants to complete their training and follow-up on time (Figure 2).

Rehabilitation physicians review patients' health data during home exercise training, and provide suggestions. If the patient's condition worsens, the system can be used to coordinate a referral to the hospital. We applied the *Xinankang* app to integrate patients, home electrical equipment, and doctors. Adherence is assessed during telephone calls and review of the participant's exercise diary.

### Cardiac Magnetic Resonance Imaging Procedure

CMR imaging is performed on a clinical Philips system (3.0 T, Philips Healthcare, Best, The Netherlands) with simultaneous ECG recording (21). RV wall thickness, stroke volume (SV), RV and left ventricle (LV) ejection fractions are obtained (21). RV/LV masses and end-diastolic volumes were obtained from the endo- and epicardial contours of the RV and LV delineated manually by blinded observers. The detailed CMR images, layers and sequences of above-mentioned parameters were available on Supplementary Material 3.

### Outcomes

#### Primary Efficacy Outcome

The changes of right ventricular stroke volume (RVSV) from baseline to Week 26 are determined by CMR. RVSV indexed for BSA is shown to predict outcome (22). RVSV quantifies blood flow independently of HR. An increase in RVSV reflects favorable, reverse remodeling of the right ventricle (RV) and is expected to reduce PAH symptoms through better oxygenation.

#### Secondary Efficacy Outcome

In the same method as that used for the primary endpoints, secondary endpoints are assessed at 26 weeks. They consist of characteristics of the RV, and of widely accepted tools to assess PAH progression:

- RV end diastolic volume (RVEDV),
- RV end systolic volume (RVESV),
- RV ejection fraction (RVEF),
- RV mass,
- WHO FC,
- 6 MWD, and
- N-terminal pro-B-type Natriuretic peptide (NT-proBNP).

Variables pertaining to the left ventricle (LV) are also assessed.

Changes from baseline to Week 26 in:

- LV SV,
- LV end diastolic volume (LVEDV),
- LV end systolic volume (LVESV),
- LV ejection fraction (LVEF),
- LV mass,
- RVEDV/LVEDV, and
- RVESV/LVESV.

#### Exploratory Efficacy Outcome

The following exploratory endpoints aim to investigate the molecular impact of exercise training on patients, including metabolomic, lipomic, and proteomic changes from baseline to Week 26 for subjects who agreed to participate in optional proteomic, metabolomic, and transcriptomic analysis, blood samples are collected at baseline and Week 26. The effects of rehabilitation exercise on proteomics, metabolomics, and transcriptomics in patients with PAH are observed. Subjects can agree to participate in all, part, or none of these analyses, and subjects who are unwilling to participate can still participate in the main clinical study.

### Study Timeline

A diagram timeline is presented in Table 2. Elaborated baseline evaluations are shown in Supplementary Material 3.

**Table 2:** Study timelines overview.

	**Screening**	**Baseline**	**Observation period**		
	****~**0 month**	**0 month**	**Week 12**	**Week 16**	**Preterm termination**
On-site clinic visit		**√**			**√**
Monthly telephone contact			**√**	**√**	**–**
Informed consent	**√**	**–**	**–**	**–**	**–**
Review eligibility criteria	**√**	**√**	**–**	**–**	**√**
Demographics	**√**	**√**	**–**	**–**	**√**
PAH history	**√**	**√**	**–**	**–**	**–**
Medical history	**√**	**√**	**–**	**√**	**√**
Body mass index	**√**	**–**	**–**	**√**	**√**
Physical examination	**√**	**√**	**–**	**√**	**√**
Vital signs	**√**	**√**	**–**	**√**	**√**
6MWD/HHR	**√**	**√**	**–**	**√**	**√**
Borg scores	**√**	**√**	**–**	**√**	**√**
Echocardiography	**√**	**√**	**–**	**√**	**√**
Right heart catherization	**√**				
Pulmonary function test	**√**				
V/Q lung scan	**√**				
CPET	**√**	**√**	**–**	**√**	**√**
WHO functional class	**√**	**√**	**–**	**√**	**√**
Electrocardiogram	**√**	**√**	**–**	**√**	**√**
Chemistry, hematology		**√**		**√**	
NT-proBNP	**–**	**√**		**√**	
Randomization	**√**	**√**	**–**	**–**	**–**
AE monitoring	**√**	**√**	**–**	**√**	**√**
Concomitant medication	**√**	**√**	**–**	**√**	**√**
Omics*	**√**			**√**	**√**

6MWD, 6-min walk distance; NT-proBNP, N-terminal pro-brain natriuretic peptide; PAH, pulmonary arterial hypertension; V/Q, ventilation-perfusion; WHO, World Health Organization; CPET, cardiopulmonary exercise testing; HHR, heart rate recovery; AE, adverse event. *Omics indicates proteomics, metabolomics and transcriptomics.

### Allocation Concealment and Blinding

A computer-generated, permuted-block randomization sequence were applied for allocation with two block sizes. The patients are randomized to the control group or receive exercise training group in a 1:1 ratio (stratification by sex). The sequence is concealed in an opaque envelope until the intervention is assigned. Patients cannot be blinded to the outcome of randomization, Furthermore, to increase the robustness of the outcomes, outcome assessors are blinded to the outcome of randomization.

### Sample Size Calculation

For CMR-based RVSV, no individual patient data are available. Previously, the REPAIR STUDY demonstrated RVSV from 52.2 ± 17.2 mL to 64.9 ± 19.0 mL (mean ± SD) after 3–5 months under PAH-specific drugs (23). The change from baseline in approximate RVSV was ~9 mL, in line with the literature suggesting that a difference of 8–12 mL is clinically relevant (24). Assuming a standard deviation of 18–22 mL, also based on the hemodynamics sub-study of SERAPHIN, the minimum sample size was 77 subjects for each group, for a total of 154 subjects with a statistical power of 90% and two-sides test of *P* < 0.05. Considering the dropout rate of 20%, the planned sample size was 192 subjects, with 96 in each group.

### Data Collection and Management

The detailed participant schedule of assessments is presented in Table 1. Demographic characteristics at enrolment, medical history, physical measurements, medication lists, and NT-proBNP are collected via an electronic medical record and interview by trained research nurses and investigators. In order to avoid missing or erroneous data, data are further verified. The research staff have only the authority to read the database.

### Data Management and Monitoring

A trial inspect will be conducted at least once a year to check research progress and protocol compliance (25). The monitoring report will be submitted to the ethics committee and investigators. The audit is available based on the monitoring report. The study did not need predetermined interim analysis. A statistician will analyze the study results.

### Adverse Events

Participants may voluntarily withdraw at any time, or if they experience a serious adverse event (SAE) that is associated with exercise training. All SAEs are discussed with the principal investigator who assesses seriousness and causality. Other adverse events (AEs), defined as any unfavorable and unintended sign, symptom or diseases, are also recorded. AEs are followed up until normalization, recovery or symptoms stabilize.

### Statistical Analysis

Data will be presented as mean (SD), median (interquartile range), and frequency (percentage) available for data characteristics. For comparisons between groups, a *t*-test or rank-sum test will be applied for quantitative variables, while the χ^2^ test or Fisher's exact test for qualitative variables.

Primary and secondary endpoints will be analyzed using the whole population. However, patients who withdrew their consent, patients with severe protocol violations, or patients without the primary endpoint data, will be excluded from the whole population (26). An intention-to-treat analysis will be used to avoid, with inclusion of all available measures, regardless of whether participants completed the intervention, while the safety analysis will be done in the safety analysis population. Primary outcome analysis will be performed by an analysis of covariance (ANCOVA) model. Changes from baseline in RVSV will be analyzed using an ANCOVA with a factor for other PAH-specific therapies (none, background, or initiated at baseline) and a covariate for baseline RVSV. Missing outcomes were imputed as the multiple imputation. For analysis of categorical data, the McNemar–Bowden test will be used. *P* < 0.05 with two-sided test is considered statistically significant. All analyses will be performed with IBM SPSS V20 (IBM Corp., Armonk, NY, USA).

### Patient and Public Involvement

Neither patients nor the public participated in the planning, implementation, analysis and evaluation of this study (26).

### Ethics and Dissemination

The study and protocol were approved by the Ethics Committee of Shanghai Pulmonary Hospital (L20–017), and registered at http://www.chictr.org.cn/index.aspx (ChiCTR2000031650). All patients will provide informed consent (Supplementary Material 4). Project results will be made publicly available in *Chinese Clinical Trial Registry* within 6 months after the trial is completed. In addition, we plan to publish the results of the protocol in a peer-reviewed journal.

## Discussion

Evidences suggests that supervised individually adjusted exercise training rehabilitation are likely to be safe for patients with PAH who are stable on PAH-targeted therapy (10, 13, 14).

Most exercise training trials published thus far have focused on exercise capacity of PH patients. There was only one study which assessed invasively measured hemodynamics changes at rest and during exercise as secondary endpoints (27). The study revealed a significant increase in the cardiac index at rest and exercise, decreases in the pulmonary arterial pressure and pulmonary vascular resistance at rest. Recent data suggest that the cardiac index during exercise may independently predict the survival of PAH patients (28). Improved RV function and hemodynamics by supervised exercise training may contribute to improved exercise capacity and QoL. Because invasive data are only available from a single perspective randomized study (27), further investigations are needed to confirm these data. Our study is the first to focus on aerobic exercise training for PAH patients in China. This prospective, randomized, controlled trial study will shed light on right ventricular properties.

In addition to assessing the efficacy of the intervention described above, this study will have several strengths. CMR-derived right ventricular remodeling parameters, RVEDV, RVESV, and RV mass, have independent prognostic value for all-cause death and composite end points of patients with PAH (25). In this study, we will first consider the RV remodeling parameters as endpoints. A combined effect on different molecular pathways and organs is likely to be the pathophysiological underpinning of the improvement associated with exercise training in PH (15). We will also investigate the proteomic, metabolomic, and transcriptomic changes after exercise training as exploratory endpoints. There were apparent changes or improvements made in patients where medication could have been adjusted but was not adjusted due to the ongoing study. This maybe the limitation of our study.

## Ethics Statement

The studies involving human participants were reviewed and approved by the Ethics Committee of Shanghai Pulmonary Hospital. The patients/participants provided their written informed consent to participate in this study.

## Author Contributions

RJ, PY, Y-QS, TS, M-YZ, and Y-MJ were involved in implementation of study protocol and analysis design. All authors contributed to data acquisition for PAH cases, with RJ, LW, J-ML, and Y-QS leading patient recruitment. RZ, Q-HZ, H-LQ, C-JL, JH, and S-GG were involved in data acquisition. RJ and LW drafted the manuscript. All authors provided critical revisions and approved the final version.

## Funding

This work was funded by the Program of National Natural Science Foundation of China (81700045, 81870042, and 82000059), the Program Supported by the Fundamental Research Funds for the Central University (22120180539), and the National Key Research and Development Project (2018YFC1313603).

## Conflict of Interest

The authors declare that the research was conducted in the absence of any commercial or financial relationships that could be construed as a potential conflict of interest.

## Publisher's Note

All claims expressed in this article are solely those of the authors and do not necessarily represent those of their affiliated organizations, or those of the publisher, the editors and the reviewers. Any product that may be evaluated in this article, or claim that may be made by its manufacturer, is not guaranteed or endorsed by the publisher.
