# Supplementary material for: A Study of the Efficacy and Safety of Aerobic Exercise Training in Pulmonary Arterial Hypertension (the Saturday Study): Protocol for a Prospective, Randomized, and Controlled Trial
Source: Front Med (Lausanne). 2022 Apr 5;9:835272. doi: 10.3389/fmed.2022.835272 (PMC9016180; doi:10.3389/fmed.2022.835272)
Supplement: Supplementary file 3 [file Data_Sheet_3.PDF]

知情同意书
(Informed consent)

试验编号：

研究方案名称：一项评估有氧运动治疗肺动脉高压患者的有效性和安全性研究

主要研究者：　姜蓉 副教授
同济大学附属上海市肺科医院肺循环科
地址: 中国上海市杨浦区政民路 507 号（邮编：200433）
电话: 86-021-65115006-3043

我们在此邀请您参加一项研究。研究的原因是评估有氧运动治疗肺动脉高压患者的有效性和安全性研究。在您决定参加本项研究之前，了解进行本项科学研究的原因及相关信息对您将非常重要。请您用一定的时间仔细阅读下面的内容，若有不清楚的问题或术语，请您与有关的医生进行讨论。如果您愿意，请与您的家人或医生进行商议。

进行研究是什么?

肺动脉高压是一种由于肺血管重构而导致肺血管阻力进行性增高、右心功能不全的疾病。在过去的十年，肺动脉高压的靶向药物有了迅速的发展。尽管靶向药物的问世极大延缓了疾病的进展，但大部分肺动脉高压患者仍表现出显著的症状，例如运动耐力及生活质量下降，甚至死亡。因此，对高效的非药物治疗需求正在迅速增长。

运动训练是最重要、最安全和最具成本效益的治疗方法之一，已被证明对多种疾病都有益。左心衰患者的康复治疗已被指南推荐，心脏康复能够改善生活质量和运动能力，降低心力衰竭相关住院风险。

心脏康复对于肺动脉高压患者也证实有裨益。越来越多证据发现对稳定期的肺动脉高压患者进行运动康复训练能够得到明显的临床获益，并在分子水平上减轻炎症和细胞增殖，改善肺血管重构。注意是，大多数肺动脉高压患者确诊时就存在右心结构和功能改变；因此心脏康复方案制定时，一定要谨慎，并做到个人化。

进行本项研究的目的是什么?

本研究目的是评估在靶向药物治疗肺动脉高压至少稳定 3 个月之后心脏康复训练（有氧运动）对改善肺动脉高压患者运动耐力的有效性及安全性。

我是否必须参加次研究?

是否参加本项研究，将由您自愿做出决定。如果您决定不参加本项研究，您不会受到任何惩罚或失去您应该拥有的任何利益。您有权在研究的任何阶段退出研究，而不会影响您的法定权利。一旦您决定参加本项研究，请您签署此知情同意书表明同意。

什么样的人不适合参加本项研究?

下列患者不适合参加本项研究：
1. 绝对禁忌症：
①急性心肌梗死（2 天内）；
②未控制的不稳定性心绞痛；
③未控制的心律失常，引发血液动力学不稳定；
④急性心内膜炎；
⑤有症状的严重主动脉缩窄；
⑥失代偿的心力衰竭；
⑦急性肺栓塞、肺梗死或深静脉血栓形成；
⑧急性心肌炎或心包炎；
⑨急性主动脉夹层；
⑩残疾人有安全隐患或不能全力完成运动试验。
2. 相对禁忌证：
①已知左冠状动脉主干狭窄；
②中到重度主动脉狭窄，与症状有不确定关系；
③心室率未控制的心动过速；

④获得性高度或完全房室传导阻滞；
⑤严重的肥厚型梗阻性心肌病（静息流出道压差高）；
⑥近期脑卒中或短暂脑缺血发作；
⑦难以合作者；
⑧静息血压>200/110mmHg；
⑨尚未纠正的临床问题，如严重贫血、电解质紊乱和甲状腺功能亢进。
3. WHO 第 3 组 PH/WHO 第 4 组 PH/WHO 第 5 组 PH

我需要做什么?

您应该按照治疗要求进行心脏康复训练（有氧运动）。
非常重要的是，在整个治疗期间您继续进行原有的肺动脉高压靶向药物及基础药物治疗。您还必须及时告诉医生您的身体状况的变化，包括健康状况变差。如果您去其他医生处或医院就诊，需通知他们您已经参加了本项临床研究，并且把病历和“知情同意书”给他们看，以确保新的医生能充分了解您的情况。还应尽快和原来的医生进行联系，告知这些事情。

本项研究我可能有什么不适或危险?

心脏康复运动可能存在的不适：心脏表现：心绞痛、心律不齐、心电图严重异常（心电图提示ST 段压低 2mm 伴有胸痛或 ST 段压低 3mm 不伴有胸痛，频发的异位心律，二度、三度的房室传导阻滞）；外周循环障碍表现：脸色苍白、皮肤湿冷、严重高血压（收缩压＞250mmHg 和/或舒张压＞120mmHg,血压较基础血压下降>30mmHg）；呼吸系统：严重呼吸困难，脉搏血氧饱和度＜80%；中枢神经系统及胃肠表现：头痛、头晕、共济失调；恶性、呕吐。与研究同时进行心电监护及动态血压监测（详见试验方案）。

其他研究相关的危险：

未知危险：
有时在研究过程中一些对研究药物有意义的新发现可能会影响您是否继续参加本项研究的决定。如果发生上述情况，医生将与您进行讨论并且由您决定是否继续研究。当然，医生有权决定您是否应终止研究。无论何种情况，研究者都会对您此后医疗作出安排。如果您仍决定继续研究，您需签署更新的知情同意书。

参加后可能获得的利益?

我们将用研究药物积极地为您治疗右心衰竭，但是不能保证您的病情一定会有所改善。研究中获得的数据将帮助医生将来更有效地治疗右心衰竭的患者。

如果不参加研究，我还有什么选择?

该研究是在原有靶向药物治疗稳定的基础上进行有氧运动，以便更好改善患者心肺功能。如果您决定不参加研究，对原有的治疗方案不会有影响，仍继续维持原有医生制定的药物治疗方案进行。

如果研究中您有任何疑问或不适应该怎么办?

______________医生将对您的整个研究阶段负责，若您出现与研究相关的疾病或伤害，或与研究相关的问题，请随时通过电话______________向他（她）或其授权的医生进行咨询。

如果研究过程中出现任何不良事件，您将得到正确和迅速的治疗。

在未经我同意的情况下，是否会被停止参加研究?

在下述情况下未经您允许，您将被停止参加研究:
- 您的安全性有很大的危险；
- 您的健康状况未得到改善或更加恶化
- 您不符合入选标准；
- 您对研究要求的依从性不足；
- 您不遵从研究者的指导；
- 研究申办方同济大学上海市肺科医院肺循环科终止研究。

我参加研究一事是否保密?

如果您决定参加研究，您的签字就意味您的病历记录可以被同济大学上海市肺科医院授权的有关人员或代表、相关权威机构、伦理委员会查阅。查阅的目的是保证整个研究信息获得的准确性和研究方案被正确的执行。能够表明您身份的记录都将进行严格的保密，除非在特殊情况下，法律或司法程序需要揭示您的身份。从本项研究中获得的资料将可能被发表，但是不包括您的姓名。

研究结果将如何应用?

研究结果将计划在国外医学期刊或相关会议、研讨会上发表。在上述情况下，您的个人资料将保密，如果您需要与此有关的详细信息，请与您的医生联系。

谁是本项研究的组织者和申办方?

同济大学上海市肺科医院肺循环科申办和组织本项研究。

谁审阅过本研究?

本项研究的详细资料已经由"同济大学附属上海市肺科医院伦理委员会"审阅。如果您需要伦理委员会的详细的资料，可从您的医生处获得。

如需了解更多的情况，请您与负责医生联系

如果您对本项研究有疑问或需要报告与研究有关的疾病或损伤，请打电话________________联系您的负责医生______________。

研究中心和您均将保留 1 份您已经签字的"知情同意书"。

感谢您参加本项临床研究！

研究编号:
研究方案名称：一项评估有氧运动治疗肺动脉高压患者的有效性和安全性研究

主要研究者: 姜蓉 副教授
同济大学附属上海市肺科医院肺循环科
地址: 中国上海市杨浦区政民路 507 号（邮编：200433）
电话: 86-021-65115006
研究中心名称: 上海市肺科医院肺循环科

知情同意书

由受试者本人或其合法代表签署：

1. 我确认已阅读并理解了此研究的知情同意书（版本日期 2020 年 01 月 11 日，第 1 版），本研究的心脏康复作用及可能发生的不良反应已经向我解释，并且我有机会提出自己的疑问。
2. 我已明确参加研究属于自愿行为，拒绝参加研究不会给我带来处罚或损害我应有的利益。同时，我了解我有权在任何时候退出研究，而不会影响到我的医疗保证或合法权利。
3. 我已得知同济大学上海市肺科医院的代表或其授权的个人，本研究相关的伦理委员会均有权审阅研究记录和病例资料，我同意上述方面的人员直接得到我的研究记录，并了解上述信息将得到保密处理。
4. 我同意参加本项临床研究。

患者：

姓名全名 ....

签字 .... 日期 ....

合法代表： (若有必要时应获得)

姓名全名 ....

签字 .... 日期 ....

由执行知情同意过程的医生完成：

我确认已就本研究的性质、目的、要求和可能的风险向患者进行了解释和讨论，并同时探讨了其它可选择的治疗方案，并确保本受试者信息的复印件已交给患者保存。

姓名全名 .... 职位 ....

签字 .... 日期 ....

由独立的见证人完成（若有必要时）

我确认受试者信息及其它书面资料中的相关信息已被准确地解释并得到受试者或其合法代表的充分理解，并得到了该受试者或其合法代表完全自愿的同意。

姓名全名 ....

签字 .... 日期 ....
