# Supplementary material for: A Study of the Efficacy and Safety of Aerobic Exercise Training in Pulmonary Arterial Hypertension (the Saturday Study): Protocol for a Prospective, Randomized, and Controlled Trial
Source: Front Med (Lausanne). 2022 Apr 5;9:835272. doi: 10.3389/fmed.2022.835272 (PMC9016180; doi:10.3389/fmed.2022.835272)
Supplement: Supplementary file 4 [file Data_Sheet_4.PDF]

The protocol of evaluations

1. Medical history

The patient's relevant medical history up to start of study (i.e., informed consent signature) must be recorded in the CRF and should include, but is not limited to:

− Complications or symptoms associated with PAH (e.g., peripheral edema or right heart failure)

− Any chronic conditions (e.g., diabetes, hypertension, angina pectoris)

− Acute conditions present at screening or which occurred in the past with sequelae

− Any serious condition in the past resolved without sequelae (e.g., acute peritonitis)

2. 6-minute walk distance/heart rate recovery

The 6MWD is a non-encouraged test which measures the distance covered over a 6-minute walk. The Borg dyspnea index is evaluated after each 6MWT. It rates dyspnea on a scale from '0' to '10'. HRR will be determined by measuring the difference between heart rate measured immediately upon completion of 6MWT and heart rate measured 1 minute after completion, preferably in a standing position, and repeated in the same manner throughout the study period.

3. WHO Functional Class

WHO FC is a classification which reflects disease severity based on symptoms.

4. Right heart catheterization

An 8 F introducer sheet was placed in the left antecubital vein or left subclavian vein, and a 7 F Swan-Ganz catheter (Edwards Lifesciences Co., Ltd, USA) was advanced into the pulmonary artery. Correct positioning of the catheter was verified by fluoroscopy. Transducers were positioned at the midaxillary line and zeroed at atmospheric pressure. mPAP, systemic arterial pressure (SAP), right atrial pressure (RAP), and pulmonary artery wedge pressure (PAWP) were measured, along with mixed venous saturation and systemic arterial oxygen saturation. Cardiac output (CO) was measured in triplicate by the thermodilution technique (Cardiac Output Computer; GE, USA) with iced normal saline. The cardiac index (CI) was calculated by dividing CO by body surface area (BSA). Pulmonary vascular resistance (PVR) was calculated as mPAP minus PAWP divided by CO. Systemic vascular resistance (SVR) was calculated as SAP minus RAP divided by

CO.

5. Blood tests

Blood samples will be taken and analyzed, including chemistry, hematology and NT-proBNP.

6. CMR

CMR Image Acquisition.

CMR imaging is performed on a clinical 3.0 T Philips Ingenia system (Philips Healthcare, Best, The Netherlands) with simultaneous ECG recording. All short-axis cine images were acquired from base to apex of the left ventricle with combined ECG gating and respiratory gating. Cine MRI was performed using the balanced steady-state free-precession (b-SSFP) sequence, with the parameters of repetition time (TR) = 2.8 ms, echo time (TE) = 1.4 ms, slice thickness = 7 mm, slice gap = 3 mm, acquired matrix = 1.2 mm × 1.2 mm, flip angle = 45°, 30 phases, field of view (FOV) = 300 mm × 300 mm.

Image post-processing.

CMR image post-processing was performed with commercial software (cvi42; Circle Cardiovascular Imaging Inc., Calgary, Canada). Short-axis cine images were imported to the Short 3D module for analysis. Endocardial and epicardial contours of LV and RV were performed manually by two radiologists who were blinded to the clinical information, cohort demographics, and RHC parameters. RV volume included the RV outflow tract portion. Both papillary muscles and trabeculations were regarded as the ventricular volume. Height and body weight were collected without shoes and heavy clothes. Individual body surface area (BSA), LV and RV end-diastolic volume (EDV), end-systolic volume (ESV), cardiac output (CO), stroke volume (SV), and ejection fraction (EF), myocardial mass (MM) were calculated by workstation software automatically, then EDV and ESV were divided by BSA, and were respectively recorded as end-diastolic volume index (EDVi), end-systolic volume index (ESVi).

7. Echocardiography

Echocardiography will be performed according to the guidelines detailed in the Echo image acquisition protocol:

- LV eccentricity index at end-diastole

- LV eccentricity index at end-systole
- Diameter of the inferior vena cava and collapse
- Tricuspid annular plane systolic excursion (TAPSE)
- Pericardial effusion
- Tricuspid peak jet velocity
- RV systolic pressure (estimated by tricuspid valve Doppler)
- Early diastolic velocity of the jet of pulmonary valve regurgitation
- End-diastolic velocity of the jet of pulmonary valve regurgitation
- Mitral E-wave velocity
- Mitral A-wave velocity
- Mitral annulus peak early diastolic velocity E’
- E/A ratio
- E/E’ ratio
- 2D RV dimension end-diastole
- Tricuspid peak annular velocity s’
- RV end diastolic area
- Right ventricular end-diastolic transverse dimension
- Right ventricular end-diastolic longitudinal dimension
- Right atrial transverse dimension
- Right atrial longitudinal dimension
- LVEDV
- LVESV
- LVEF
- RVEDV/LVEDV

8. Electrocardiogram

The 12 lead ECG results of the subjects will be obtained, in which the subjects will take the semi recumbent position and rest for at least 5 minutes before recording. The ECG shall include lead II as a cardiogram and contain at least 5 QRS complexes. The collected ECG parameters (after at least 5 minutes of rest) included heart rate, PR interval, QT interval, QRS duration and any clinically significant abnormalities.

9. Vital signs

Vital signs will be assessed during screening, baseline, preterm termination, and Week 26.

10. Omics

Routine medical blood tube (EDTA anticoagulation) was used to collect 2 mL of fasting elbow venous blood from the subjects in the morning, which was immediately stored at 4℃. After 30 min of standing, the blood was centrifuged at 3 000 r/min for 20 min. The plasma was extracted, divided and stored in ultra-low temperature refrigerator at 80℃ for future use. The frozen plasma was taken out before the experiment and thawed at 4℃. (1) Proteins were extracted and measured by TCA/acetone precipitation method and BCA method respectively, followed by trypsin digestion and peptide discharge. Finally, LC-MS /MS method was used for proteomics study. (2) Metabolomics analysis was performed using UPLC/Q TOF MS analysis. (3) Total plasma RNA was extracted, RNA purity and concentration were determined, and cDNA was synthesized by reverse transcription reaction. Ion Proton sequencer was used to conduct high-throughput sequencing of samples for metabolomics study.
